# Concomitant septic arthritis of the hip joint and femoral head avascular necrosis in patients with recent COVID-19 infection: a cautionary report

**DOI:** 10.1186/s13018-022-03192-4

**Published:** 2022-06-06

**Authors:** Mohammad Vahedian Ardakani, Sara Parviz, Ehsan Ghadimi, Zahra Zamani, Mohammadreza Salehi, Mohammad Ayati Firoozabadi, S. M. Javad Mortazavi

**Affiliations:** 1 Joint Reconstruction Research Centre, Tehran University of Medical Sciences, End of Keshavarz Blvd, Tehran, 1419733141 Iran; 2 Advanced Diagnostic and Interventional Radiology Research Center (ADIR), Department of Radiology, Medical Imaging Center, Tehran University of Medical Sciences, Tehran, Iran; 3 Community Medicine Department, Tehran University of Medical Sciences, Tehran, Iran; 4 Imam Khomeini Hospital Complex, Infectious Diseases Department, Tehran University of Medical Sciences, Tehran, Iran

**Keywords:** COVID-19, Corticosteroid, Septic arthritis, Avascular necrosis, Total hip arthroplasty

## Abstract

**Purpose:**

At present, concomitant avascular necrosis (AVN) of femoral head and septic arthritis (SA) as a sequel of COVID-19 infection has yet not been documented. By large-scale use of life-saving corticosteroids (CS) in COVID-19 cases, our aim is to warn of the occurrence of hip joint infection in these patients.

**Methods:**

We report a series of five cases in which patients developed septic arthritis concomitant with AVN after being treated for COVID-19 infection. The mean dose of prednisolone used in these cases was 1695.2 mg. The time period of onset of hip symptoms in our cases from the beginning of the COVID-19 infection was 56 days in the first case, 43 days in the second case, 30 days in the third case, 29 days in the fourth case and 50 days in the last case, with an average time of 41.6 days. All patients underwent surgery depending on the extent of articular cartilage damage by direct anterior approach.

**Results:**

Clinical and laboratory symptoms improved significantly in all patients. The mean visual analogue pain score of the patients decreased from 9.4 (9–10) before surgery to 2.8 (1–4) after 1 week of operation.

**Conclusion:**

In any patient with the history of COVID-19 infection specially those who have been treated with corticosteroid as one of the medications prescribed during the disease, any joint symptom specially in the hips should draw our attention to the joint infection, and with timely diagnosis and surgery, their hip joint can be saved.

## Introduction

In December 2019, a cluster of severe respiratory infection was reported in Wuhan, Hubei Province, China. In January 2020, the first case of death from the virus was reported in China. Positive case reports from other countries such as Thailand, Japan, South Korea and USA have made things worse. The result of this great epidemic is filling hospital beds, excessive fatigue of medical team, severe lack of personal protective equipment, infection of hospital staff, lack of manpower and the spread of disease and anxiety of the people of the countries of the world.

Numerous evidences show that COVID-19 can affect different human body organs as a part of ‘Long COVID-19’ such as Guillain–Barre syndrome, lung fibrosis, pulmonary emboli, cardiomyopathy, skin and joints, sensory dysfunction and stroke [1, 2].

‘Long COVID-19’ is a term used to describe symptoms in patients that continue for weeks or months after recovery from COVID-19 [2].

Some cadaveric studies confirm the presence of intravascular thrombosis and coagulopathy in patients with COVID-19 infection [3–5] which can be one of the causes of femoral head avascular necrosis in these patients. Numerous cases have been reported with a diagnosis of avascular necrosis or reactive arthritis following COVID-19 infection [6–10]. All of these patients were treated with a dose of CS as part of the COVID-19 treatment regimen.

To date, no cases of purulent joint infection due to COVID-19 infection have been reported, and in particular the association of septic arthritis with avascular necrosis has not been reported. In the continuation of this article, we report five cases with septic arthritis of hip joints with some degree of AVN of femoral head following recovery from COVID-19 disease. They all received CS (methylprednisolone, prednisolone, dexamethasone) during their COVID-19 disease period along with other antiviral medications. All these cases were negative for antinuclear antibodies, rheumatoid factor, anti-cyclic citrullinated peptide antibodies, hepatitis B virus surface antigen, anti-hepatitis C virus antibodies and anti-human immunodeficiency virus antibodies. Examination of joint fluid for crystals was also negative in all cases.

## Materials and methods

### Case 1

**Fig. 1:**
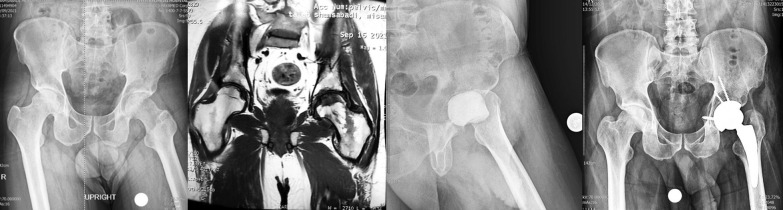
**a** Case one, preoperative X-ray, left hip involvement. **b** MRI of the hips shows left side inflammation and cartilage destruction. **c** Early post-op after first stage of arthroplasty. d) Post-op radiography, after 2nd stage

A 34-year-old male patient was diagnosed with COVID-19 on June 22, 2021. During his hospitalization, the patient was administered intravenous (IV) dexamethasone 16 mg per day for 10 days and 250 mg IV methylprednisolone in one dose and IV remdesivir and sub-cutaneous enoxaparin. Total steroid received by the patient was 1384.5 mg of prednisolone equivalent. After 56 days from the onset of COVID-19 disease, the patient developed rapidly increasing pain in the left hip joint. His laboratory (ESR 110 mm/h, CRP 140 mg/l) and radiological findings indicated an infectious process with severe cartilage damage in his left hip joint; Bone marrow abnormal signal was seen in the femoral head and serpiginous lines of abnormal signal in the superior and medial aspect of the femoral head suggestive for avascular necrosis, extensive bone marrow edema in the acetabular bone, femoral head and neck extends to the adjacent soft tissue and proximal thigh muscles which are not common in the process of avascular necrosis without infection. Aspiration was performed without US guide in supine position and under light sedation from anterior part of greater trochanter to the femoral head and it was dry. Therefore, he underwent first stage of total hip arthroplasty (THA) No clear puss was seen during the surgery and the articular cartilage of the femoral head was severely damaged and acetabular cartilage was very soft and damaged less than femoral head. The head and neck of the femur were removed and after a brief ream of acetabular cavity antibiotic cement was placed in place. Intraoperative cultures were negative for this patient. He was treated with intravenous vancomycin 1gr BID and imipenem 1gr TDS for 4 weeks with the consult of an infectious disease specialist. After 2 months when the inflammatory markers became negative, the second stage of surgery was performed. Both stages of surgery were done under spinal anesthesia. At the time of submitting this article for publication, 3 months have passed since surgery and he lives a painless life (Fig. 1).

### Case 2

**Fig. 2:**
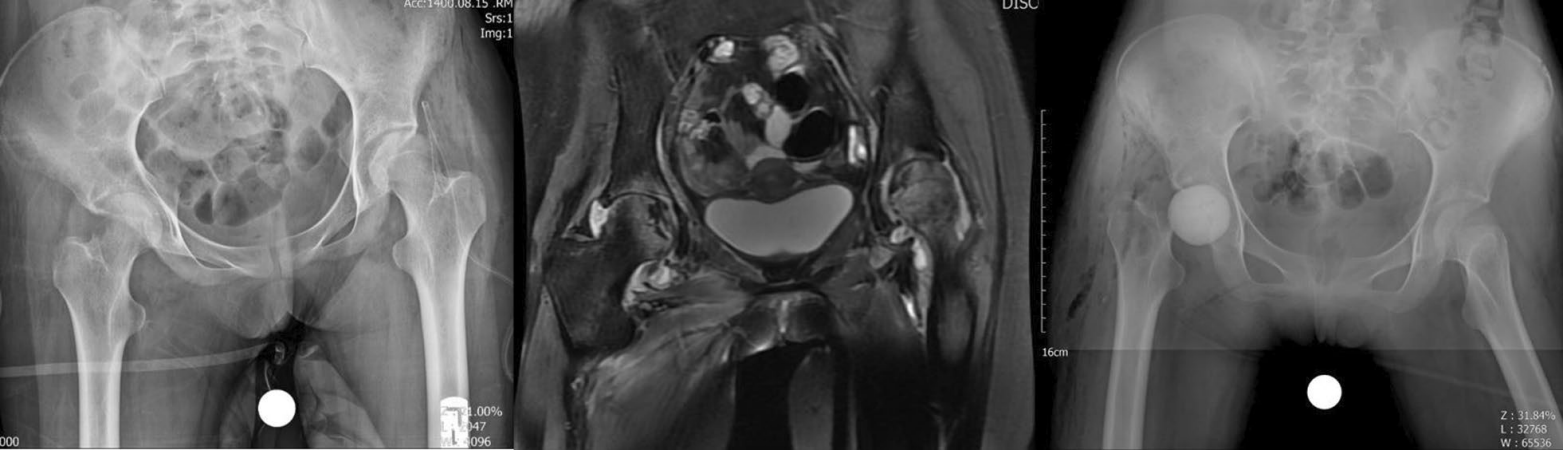
**a** Case two, preoperative X-ray, bilateral involvement. **b** MRI of both hips shows cartilage destruction on right side and acute inflammation on left side. **c** Post-op radiography after first stage of THA for right side and arthrotomy and irrigation and debridement for left side

A 14-year-old female patient known case of ALL was diagnosed with COVID-19 on July 18, 2021, for which IV remdesivir and IV methylprednisolone (750 mg) and 16 mg dexamethasone per day for 10 days were given equivalent to 2009.5 mg prednisolone. One month after being diagnosed with COVID-19 disease she contracted salmonella bacteremia and 43 days post-COVID-19 detection she developed pain in the bilateral groin that was very increasing. The pain was more severe on the right side. Laboratory markers indicated an inflammatory process (ESR 64 mm/h, CRP 90 mg/l) and radiological findings (MRI with gadolinium enhancement) indicated destruction of the right articular cartilage and active infection without cartilage damage in the left hip joint; there was bone marrow edema of bilateral femoral head which extended to the femoral neck on the right side. Edema was extended along the abductor muscle groups bilaterally and an enlarged pelvic lymph node was seen on the right side. Aspiration of both hips was done without US guide. Clear pus was aspirated on the right side and turbid fluid with slight debris on the left side. Non-typhoidal salmonella group D grew on both sides of the hip aspiration. Finally, she underwent arthrotomy and irrigation and debridement through the direct anterior approach (DAA) for left side and the first stage of two-stage THA also via DAA for the right side. Surgeries were done under spinal anesthesia, simultaneously. Our technique for the first stage of arthroplasty was the same in all patients. Articular cartilage of the left hip was intact but severe destruction of femoral head and acetabular cartilage was evident on right side. Intraoperative cultures of salmonella were also reported for both sides. The initial treatment included 14 days of intravenous ceftriaxone (1 g daily) before step down to oral ciprofloxacin 500 mg/BID/PO for 6 weeks. Antibiotic treatment was performed under the supervision of an infectious disease specialist. After 6 weeks of surgery, the laboratory markers were negative and there was no pain or any other symptom on the left side. She was waiting for the second stage of THA for the right side, unfortunately her blood disease was activated and she is currently under the supervision of a hematologist and the permission for the second stage THA has not been issued, yet (Fig. 2).

### Case 3

**Fig. 3:**
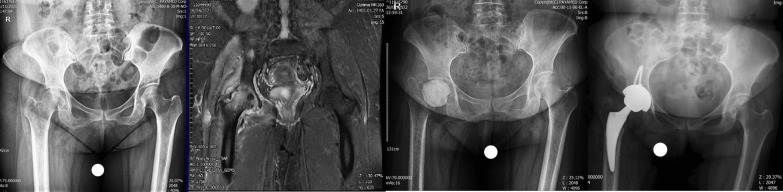
**a** Case three, preoperative X-ray, right side involvement. **b** MRI of the hips showing right side joint destruction. **c** Post-op radiography after first stage of arthroplasty. **d** Post-op radiography after second stage of THA

A 52-year-old woman was diagnosed with COVID-19 on April 25, 2021. The patient was given oral dexamethasone 25 mg per day for 8 days and in tapering dose over 10 days (total 300 mg equivalent to 2010 mg of prednisolone). She was known case of breast cancer and underwent 30 sessions of radiotherapy and several rounds of chemotherapy, last time one year ago. Thirty days after the COVID-19 diagnosis the patient began to have increasing pain in her right hip joint. Laboratory findings indicated an inflammatory process (ESR 40 mm/g, CRP 69 mg/l) and radiological findings revealed hip joint infection and osteomyelitis; classic findings of avascular necrosis along with edema and inflammation in the peri-articular soft tissue including the adductor, abductors and gluteal muscle groups, signal changes of the bone on the both sides of the joint and enlarged pelvic lymph node which are not routinely seen in avascular necrosis. The hip joint aspiration without US guide was done and 3 cc turbid fluid was obtained. Cultures were reported to be negative. The decision for two-stage THA surgery was made for her and eventually she underwent first stage of arthroplasty under spinal anesthesia and through DAA; abundant effusion was seen and articular cartilage of femoral head and acetabulum were severely destructed and the bone of femoral head and neck were very soft and did not have the natural consistency. The head and neck of the femur were removed like other cases and a brief acetabular ream was performed and antibiotic cement was applied to the site. Intraoperative culture results were also negative; She was being treated with vancomycin 1gr BID/IV and imipenem 1gr/TDS/IV for 4 weeks with the guidance of infectious disease specialist. After 6 weeks the laboratory markers became negative and after another 2 weeks and markers remained negative, the second stage of THA was performed for her via DAA. Intraoperative cultures at this stage were negative. At the time of publication of this article 3 months have passed since her arthroplasty and she does not mention any hip pain or discomfort (Fig. 3).

### Case 4

**Fig. 4:**
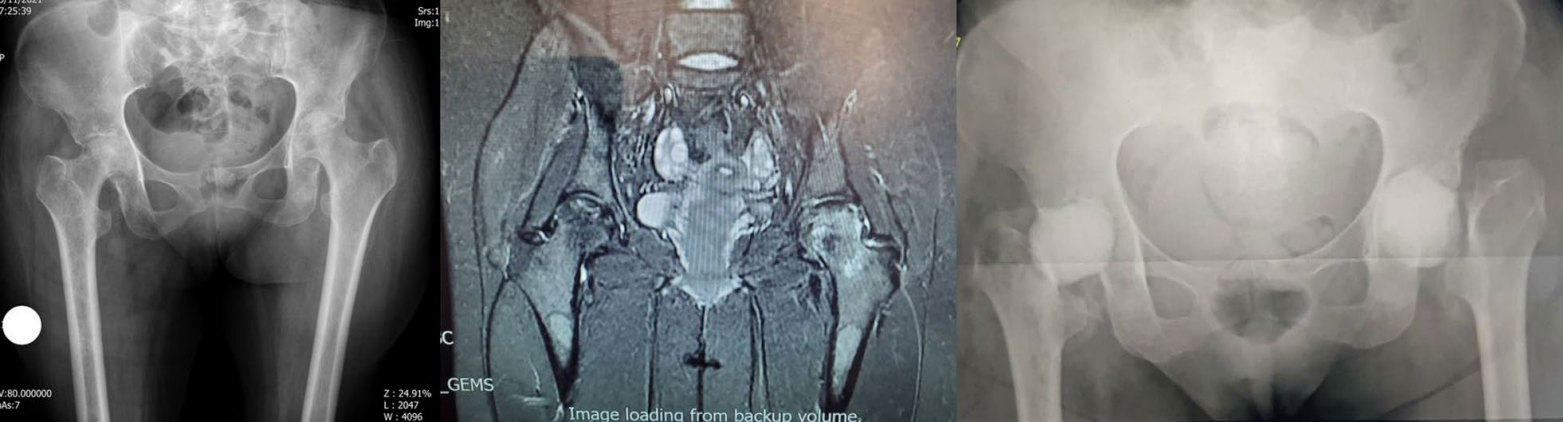
**a** Case four, preoperative X-ray, both sides involvement. **b** MRI of the hips showing bilateral joint destruction and cartilage loss. **c** Post-op radiography after first stage of THA for both sides

A 54-year-old woman patient presented to the emergency department with a history of fever and weakness and dry cough on April 2, 2021, and was diagnosed with COVID-19 infection. She completed ten-day course of dexamethasone (16 mg daily equivalent to 1072 mg of prednisolone) as well as a 500 mg dose of IV methylprednisolone. The total dose of CS prescribed for her was equivalent to 1697 mg prednisolone. Hydroxychloroquine and azithromycin were administered too. Twenty-nine days after the diagnosis of COVID-19 both hips pain and flexion contracture were developed that were rapidly increasing. Laboratory markers indicated the presence of inflammation in her hip joints (ESR 59 mm/h, CRP 65 mg/l) and radiological evidence (MRI with gadolinium enhancement) indicated that there was infection of both hip joints with underlying AVN Ficat grade 4; Bone marrow abnormal signal was seen in the femoral head and serpiginous lines of abnormal signal in the superior and medial aspect of the femoral head suggestive for avascular necrosis. There was joint effusion on both sides as well as edema of adductors and gluteal muscle group, also the effusion was seen in the sub gluteus maximus bursa, bilaterally. The large iliopsoas bursa delighted with internal debris of dimensions 68 × 48 × 100 mm on the right side and dimensions 12 × 16 × 75 mm on the left side. A number of small lymph nodes were observed in the iliac chain on both sides. Bone signal changes were seen in the acetabulum and proximal femur on both sides extending to the right femoral neck. Articular cartilage destruction and collapse of the upper femoral head portion were seen on both sides. Aspiration culture of both hips was done without US guide and Clear pus was obtained from both hip joints. Serratia marcescens grew in culture media on both sides. The first stage of two-stage THA and cement placement was performed for both hip joints in two sessions under spinal anesthesia via DAA. After opening the joint capsule some pus came out from both sides. The articular cartilage of femoral head and acetabulum was severely damaged on both sides and the bone of proximal femurs was not of adequate consistency. The head and neck of the femur were removed on both sides and after a brief ream of acetabular cavity antibiotic cement was placed in place. Intraoperative cultures also confirmed the presence of Serratia marcescens on both sides. She was treated with vancomycin 1gr/BID/IV and imipenem 1gr/TDS/IV for 6 weeks with the opinion of infectious disease specialist. Clinical symptoms have improved a lot and laboratory markers became negative after 6 weeks and she is waiting for the second stage of the surgery (Fig. 4).

### Case 5

**Fig. 5:**
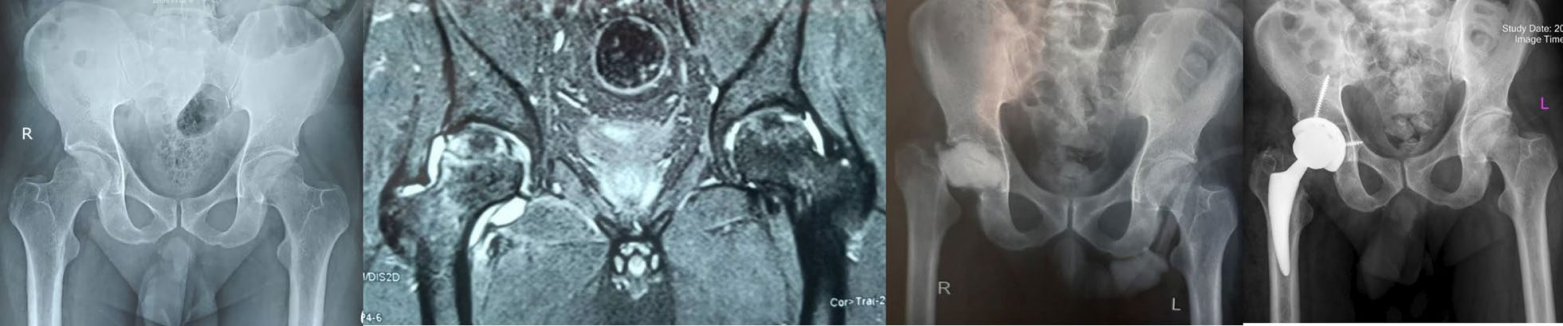
**a** Case five, preoperative X-ray, both sides involvement. **b** MRI of the hips shows right side cartilage destruction and less involvement of the left side. **c** Post-op radiography after first stage of right side and arthrotomy and irrigation and debridement of left side. **d** Final radiography after second stage THA of right side

A 38-year-old male patient was diagnosed with COVID-19 on August 4, 2021. Ten days later, the patient was admitted to intensive care at another hospital because of pulmonary involvement and dropping saturation. During his hospitalization, the patient received IV methylprednisolone 75 mg per day for 12 days (total dose of 900 mg of methylprednisolone equivalent to 1125 mg prednisolone) and IV remdesivir and IV Actemra. After discharge, oral prednisolone was administered in tapering dose for 20 days (total dose of 250 mg). The total steroid received by the patient was 1375 mg of prednisolone equivalent. Fifty days after the COVID-19 diagnosis, the patient developed pain in the right groin. A few days later, fever and pain in the left groin started. Radiograph and MRI of hips done (67 days since COVID-19 diagnosis) and showed bilateral hip AVN (Ficat-Arlet stage two on both hips) (Fig. 5).

The patient was admitted for bilateral core decompression surgery in another hospital. Fever, constitutional symptoms, elevated ESR (112 mm/h) and CRP (145 mg/l) make the previous diagnosis to be suspected. Though the planned surgery was canceled, the patient was referred to our clinic for further evaluation. Radiographic findings showed bilateral serpiginous abnormal signal line in the femoral heads compatible with avascular necrosis. Moderate joint effusion was seen in the right hip joint with bone marrow edema of the femoral head and adductor muscles. These findings were suggestive for associated septic arthritis. Mild possibly reactive joint effusion was also seen in the left hip joint. With suspicion of an infectious process, bilateral hip arthrocentesis was done without US guide. The specimens were purulent, and coagulase-positive staphylococcus grew up in the specimens of both sides. After diagnosis of bilateral SA of the hips, bilateral hip arthrotomy and irrigation and debridement was done under spinal anesthesia via DAA. After opening the capsule, some clear pus was removed. Intraoperative cultures confirmed the presence of coagulase-positive staphylococcus on both hip joints. Irrigation was performed with 9 L of normal saline for each side. He was treated with vancomycin 1gr/BID/IV for 4 weeks under the supervision of an infectious disease specialist. Despite a period of improvement in the symptoms, the pain in the right hip continued and an X-ray showed severe hip destruction in the right side. Laboratory markers increased again (ESR 85 mm/h, CRP 92 mg/l), radiographic findings at this stage were destruction of articular cartilage and signal changes were evident in the bone of the acetabular side and femoral head and neck compatible with osteomyelitis. We decided to perform first stage of two-stage THA. Extensive destruction of articular cartilage of the femoral head and acetabulum was observed along with softening of the femoral head. No clear pus was seen at this stage. The head and neck of the femur were removed according to the technique used in other cases and antibiotic cement was placed in place. After the second surgery, the laboratory and clinical findings improved dramatically. The patient was discharged with IV targocid for 6 weeks (400 mg/stat/IV and 200 mg/daily/IV) under the guidance of infectious disease specialist. Improvement in clinical and laboratory tests persisted. After 2 weeks of discontinuation of antibiotic and negative ESR and CRP, the second stage of THA was performed for him. Both stages of THA were performed under spinal anesthesia and through DAA. Intraoperative cultures at this stage were negative. In the final 2-month follow-up that we have from the patient, he does not feel pain and discomfort in the hip joints on both sides.

### Imaging findings

Preoperative imaging was done for all patients including a standard pelvic X-ray (antero-posterior view) and also pelvic and hip MRI with a standard protocol. In two cases MRI with gadolinium was performed (case two and case four).

**Fig. 6:**
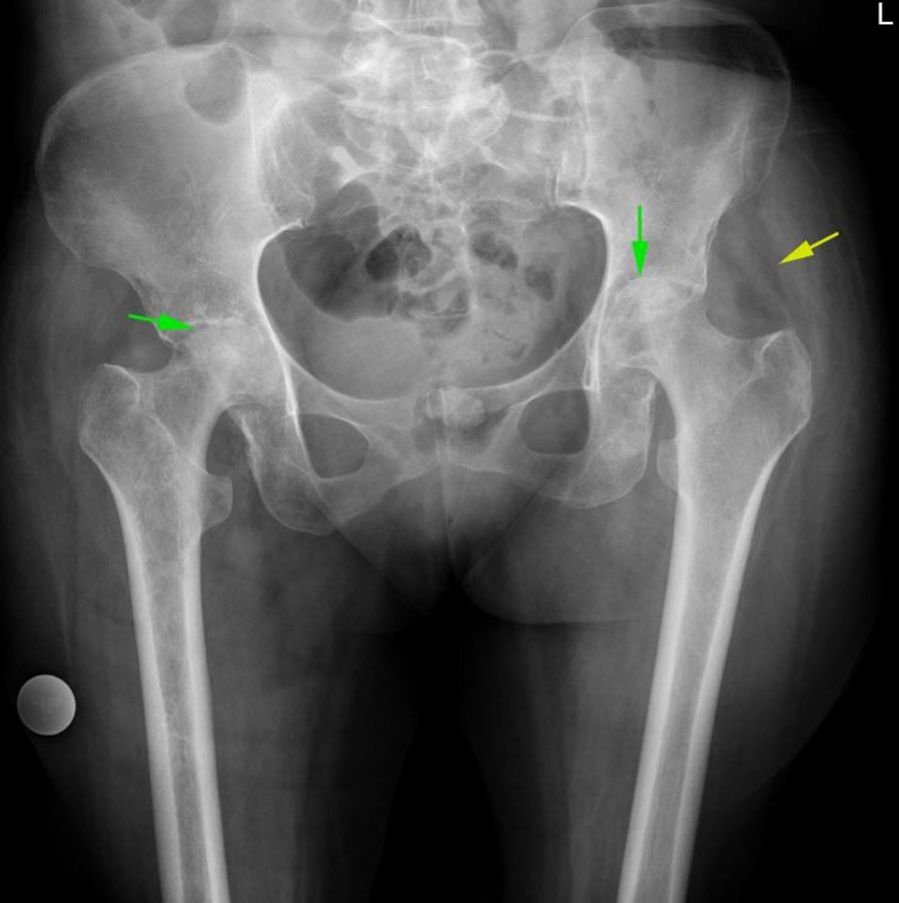
AP view pelvic X-ray shows sclerosis and collapse of bilateral femoral head in favor of femoral head AVN (green arrow). Soft tissue edema and displaced gluteal fat plans are in favor of joint fluid and possible inflammatory process (Yellow arrow)

X-ray evaluation shows sclerosis and collapse of femoral head compatible with classic avascular necrosis. An X-ray clue for the presence of an intra-articular inflammatory process was displaced gluteal fat plans. Joint space narrowing was seen in cases with cartilage damage (Fig. 6).

**Fig. 7:**
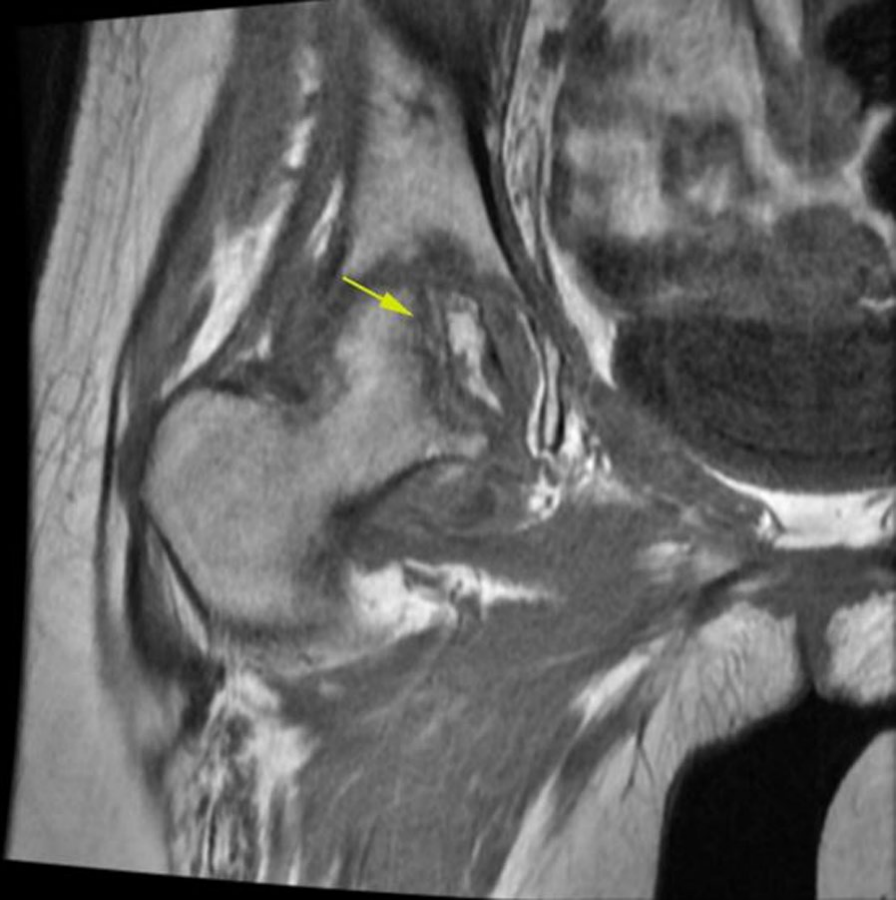
Coronal T1 non-fat sat MRI shows low signal serpiginous lines of femoral head compatible with femoral head AVN (yellow arrow).

In the MRI evaluation, all patients show classic findings of femoral head AVN as bone marrow edema and serpiginous lines of abnormal signal in the superior and medial aspect of the femoral head (Fig. 7).

An additional finding was the presence of joint effusion in the involved hip joint. Mild reactive effusion could be seen in association with the femoral head AVN especially in the advanced stage due to the superimposed degenerative changes.

**Fig. 8:**
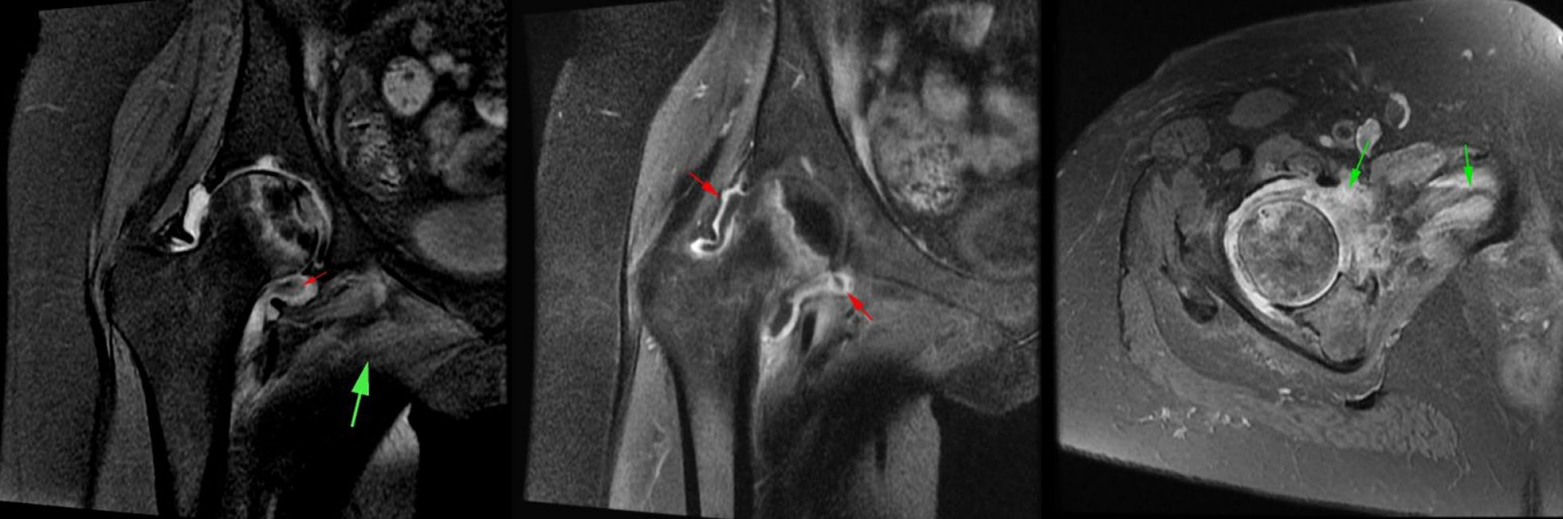
**a** Coronal PD fat sat images show femoral AVN in association with joint effusion and synovial thickening (small red arrow). There is also edema in the adductor muscle group (large green arrow). **b** Coronal T1 fat sat images in the same patient as (**a**) shows synovial thickening and enhancement after contrast injection (red arrow). **c** Axial oblique PD fat sat images in another patient shows edema in the pectineus muscle and also inferior capsule of the hip joint (green arrow).

In the cases included in our study, the amount of joint effusion was more than expected and was associated with synovial thickening and enhancement. An important clue was edema and inflammation in the peri-articular soft tissue including the adductor and gluteal muscle groups, which were not routinely seen in the femoral head AVN (Fig. 8).

**Fig. 9:**
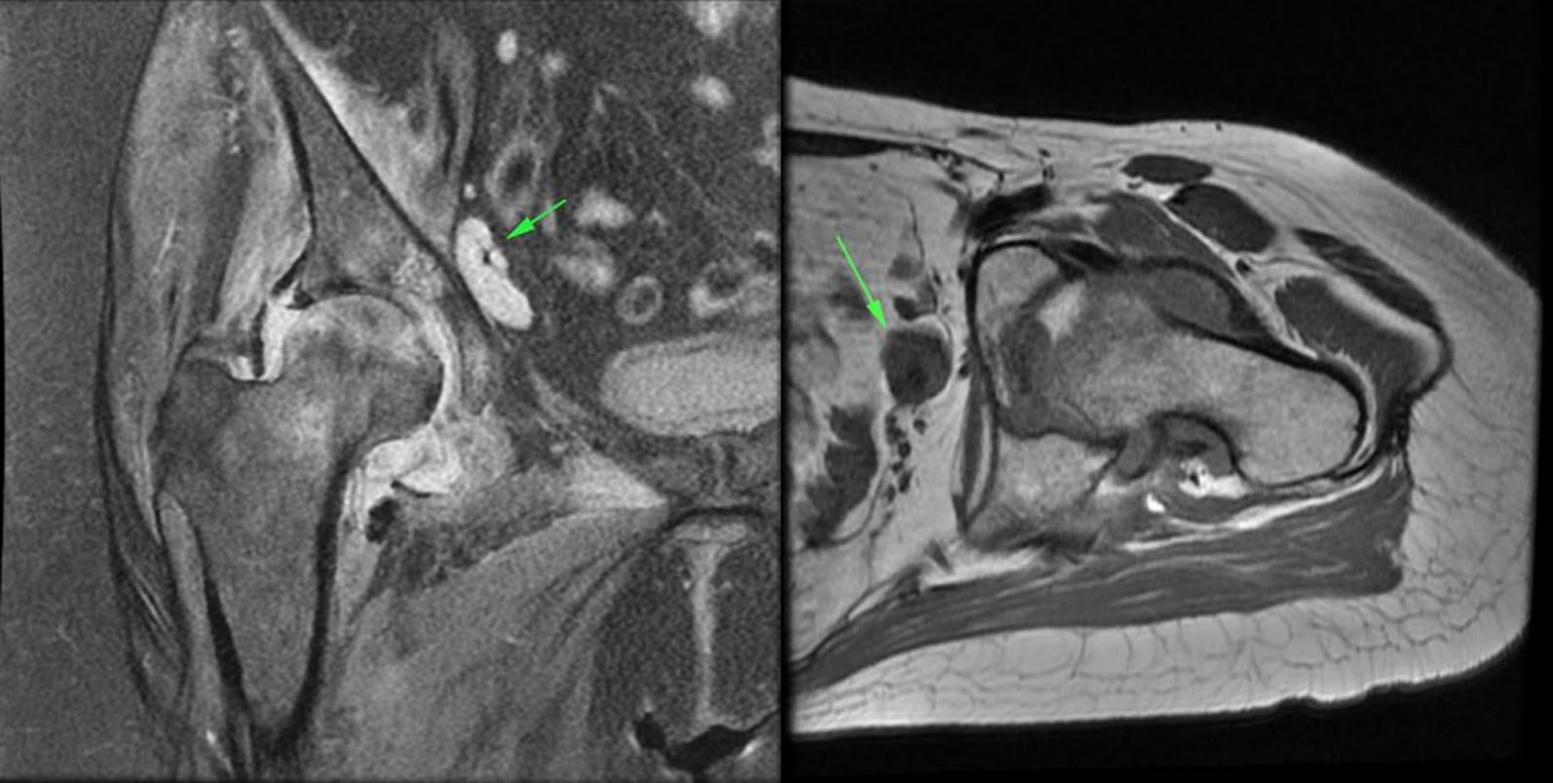
**a** Coronal PD fat sat and **b** axial T1 non-fat sat images show two different patients with enlarged lymphadenopathy in the external iliac chain (green arrow).

Another sign of inflammation was the presence of pelvic enlarged lymph nodes ipsilateral to the involved joint (Fig. 9).

**Fig. 10:**
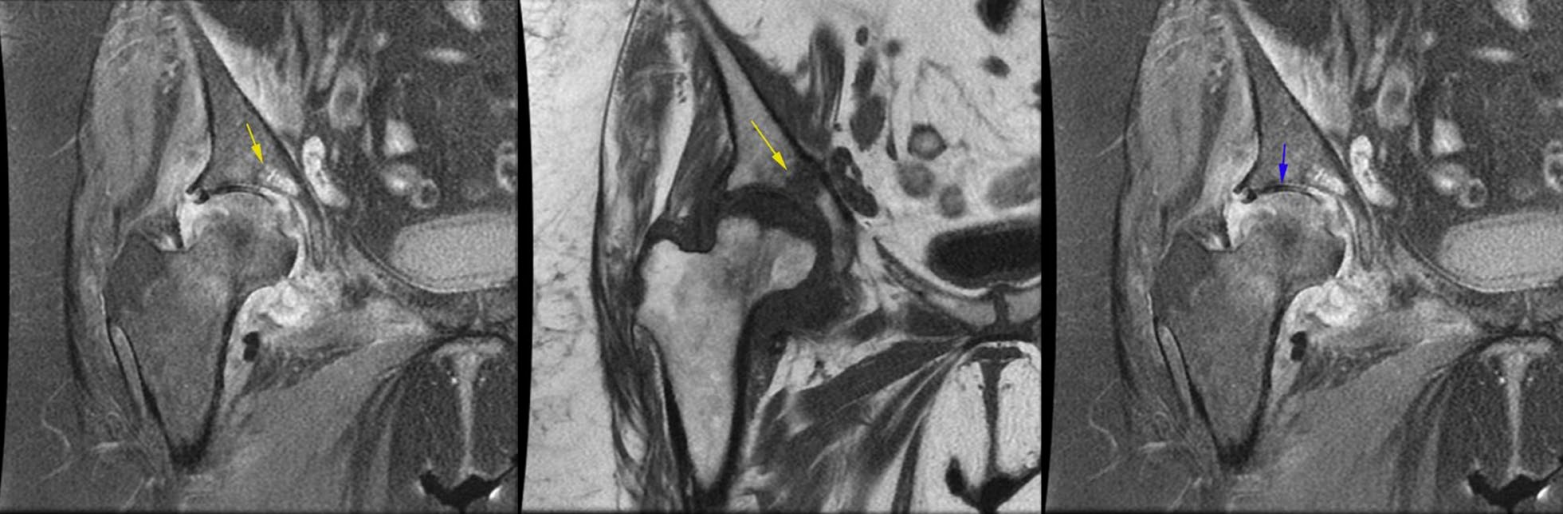
**a, b** Coronal PD fat sat image shows a focus of bone marrow edema in acetabulum which has low signal in coronal non-fat sat T1 images with signal intensity darker than adjacent muscle (yellow arrow) which was suspicious for presence of osteomyelitis, **c** coronal PD fat sat image shows joint space narrowing and full thickness cartilage loss in the weight-bearing surface (blue arrow)

Regarding these findings associated septic arthritis was suggested and joint aspiration was done. In addition, in two of the cases, a small area of abnormal signal was found in the acetabulum bone marrow suspicious for juxta-articular osteomyelitis (Fig. 10a, b).

In the cases with prolonged symptoms (all cases), cartilage damage and superimposed degenerative changes were developed (Fig. 10c).

## Results

The average dose of prednisolone equivalent steroid our patients received was 1695.2 mg (1375–2010 mg). The time period of onset of hip symptoms in our cases from the beginning of the COVID-19 infection was 56 days in the first case, 43 days in the second case, 30 days in the third case, 29 days in the fourth case and 50 days in the last case, with an average time of 41.6 days. All patients underwent surgery depending on the extent of articular cartilage damage by direct anterior approach and their clinical and laboratory symptoms improved significantly. The mean visual analogue pain score of the patients decreased from 9.4 (9–10) before surgery to 2.8 (1–4) after 1 week of operation. All of our cases experienced more severe pain than other cases of hip joint inflammation in other studies (AVN, rheumatoid arthritis, viral arthritis, reactive arthritis) before surgery. Table 1 lists the demographic characteristics of the patients as well as other factors influencing the study.

**Table 1:** Specifications of the cases reported in the study

	Sex	Age	The onset of hip symptoms (days from COVID infection)	Pre-op ESR (mm/h)	Pre-op CRP (mg/l)	Hip involved	Culture	Medication used in addition to corticosteroid	Dose of corticosteroids (mg of prednisolone)	AB used	Pre-op VAS score	Post-op VAS score
Case 1	M	34	56	110	140	Left	Negative	Remdesivir	1384.5	Imipenem + Vancomycin	10	4
Case 2	F	14	43	64	90	Bilateral	Non-typhoid salmonella	Remdesivir	2009.5	Ceftriaxone	9	1
Case 3	F	52	30	40	69	Right	Negative	Azithromycin	2010	imipenem + Vancomycin	9	3
Case 4	F	54	29	59	65	Bilateral	Serratia marcescens	Azithromycin, hydroxychloroquine	1697	imipenem + Vancomycin	10	4
Case 5	M	38	50	112	145	Bilateral	Coagulase negative staphylococci	Remdesivir, Actemra	1375	Targocid	9	2

*VAS* visual analog scale

## Discussion

Joint symptoms have been seen in a number of patients with COVID-19 infection during the recent pandemic event. These joint involvements can have different etiologies and occur in different joints. For example, drugs used to treat COVID-19 infectious disease such as CS can have side effects on the hip joints and several cases of femoral head AVN following treatment of COVID-19 disease have been reported in individuals treated with CS. Agarwala and colleagues reported three cases of AVN of femoral head following recovery from COVID-19 disease in May 2021; all of them were treated conservatively and their joint symptoms improved significantly [1]. These reports indicate the onset of AVN of femoral head in COVID-19 patients was associated with lower doses and shorter duration of CS administration compared to other patients with avascular necrosis of femoral head who did not have COVID-19 disease [11, 12]. In addition, endothelial markers were found to have elevated in the blood of critically ill patients with COVID-19 [13]. The autopsy of many of these patients confirmed endothelial dysfunction [14]. This endothelial destruction can trigger the pro-inflammatory and pro-coagulant pathways [15] and can lead to generalized microcirculatory dysfunction and related microthrombi [14] which could potentially be one of the causes of femoral head avascular necrosis in patients with COVID-19 infection.

Reactive arthritis is another form of joint complication caused by different type of infections, which can be related to mucosal infections in different areas of body such as urogenital (chlamydia) and gastrointestinal (campylobacter, salmonella, shigella, clostridium difficile, Yersinia) and respiratory pathogens (chlamydia pneumonia) [2, 16, 17]. Its incidence is reported to be 1–1.5% in gastrointestinal infections and 4–8% after urogenital tract infections [16]. People with HLA-B27 allele or family history of spondyloarthropathies are more at risk of developing reactive arthritis [16]. Most cases of this kind of arthritis are seen in the lower extremities and this is considered as a diagnostic major criteria for the diagnosis of reactive arthritis [17]. The prognosis is favorable in most cases and spontaneous improvement is seen in most cases within 6–12 months [16]. Cases of reactive arthritis associated with COVID-19 disease have been reported in lower limbs such as knees, ankle, metatarsophalangeal and interphalangeal joints, recently [2, 7, 9, 10]. A case of reactive arthritis following COVID-19 disease in the wrist and shoulder has also been reported [2].

Another cause of joint inflammation is viral arthritis which sometimes difficult to detect. It is considered in patients with acute onset poly-articular symptoms. A wide range of viral infections such as parvovirus-B19, hepatitis B, HCV, HIV, alphaviruses, HTLV-1, arboviruses, flaviviruses can cause arthritis [18]. It is specially considered in patients who have a history of traveling to certain areas.

Reactive arthritis in different from viral-related arthritis, however in the cases of COVID-19 infection recognizing these differences and naming them requires further studies. It should be noted that bacterial infections of body organs, immunological and genetic factors including HLA-B27 play an essential role in the development of reactive arthritis [19]. Accurate viral diagnostic tests as well as low-titers autoantibodies such as rheumatoid factor and antinuclear antibody can help differentiate viral arthritis from a reactive one [18]. However, both treatments are supportive and anti-inflammatory medications should be considered as treatment.

However, the most destructive type of arthritis is infectious arthritis, the early diagnosis of which, is very important. Joint function is irreversibly lost in 25–50% of cases of infectious arthritis [20]. Despite advances in antibiotics and surgical procedures, the case fatality rate of septic arthritis in the past 25 years has not changed, ranging from 5 to 15% [21, 22]. The incidence of SA varies from two to ten per 100,000 population to 30–70 per 100,000 people with rheumatoid arthritis or prosthetic implants [21].

Bacteria that most often cause SA include: staphylococcus aureus, all types of streptococci, all gram-negative bacilli, neisseria gonorrhoeae and anaerobes [21, 22] and the most common risk factor underlying that are: rheumatoid arthritis or osteoarthritis, joint prosthesis, low socioeconomic status, intravenous drug abuse, alcoholism, diabetes, previous intra-articular CS injection and cutaneous ulcers [22].

Diagnosis of SA is based on physical examination of the patient as well as laboratory markers and radiological evidences. Joint fluid aspiration is helpful in 50–67% of cases [23]. Fever, redness, swollen and very painful joint movement are common symptoms and raised WBC and erythrocyte sedimentation(ESR) and C-reactive protein(CRP) in peripheral blood are other diagnostic factors, but these tests are very non-specific. Radiological evidences including joint effusion and adjacent lymphadenopathy as well as juxta-articular osteoporosis and bone erosions and osteomyelitis are helpful in prolonged cases. Finally, destruction of articular cartilage on X-rays and MRI is evidence of prolonged joint infection [21–23]. Differential diagnoses of SA include gout and pseudo-gout, reactive arthritis, rheumatoid arthritis, lyme disease and viral arthritis, which can be greatly differentiated by accurate history and complete examination as well as various laboratory tests [21].

The most appropriate treatment for septic arthritis is joint drainage and administration of appropriate antibiotics based on the culture results and anti-biogram [21, 22]. Complete removal of necrotic and infectious material is mandatory. Joint drainage methods include closed drainage, arthroscopic drainage and open drainage specially in hip joints [22]. Appropriateness and adequacy of antibiotic type and dose and duration are determined in the literature and it should cover the most common pathogens (staphylococcus aureus and streptococcus). In patients with the history of recent inpatient, intensive care unit and other risk factors for MRSA, antibiotic regimen should consist of vancomycin with or without second or third generation cephalosporin and in patient with high risk of gram-negative sepsis (elderly, UTI, catheters) it must include second and third generation cephalosporin with or without fluoroquinolones [21, 22]. Consultation with infectious disease specialist is strongly recommended.

Studies have revealed an association between HLA (Human Leukocyte Antigen) and pathogenicity and severity as well as COVID-19 morbidity in people. Recently, Migliorini and his colleagues reviewed various studies in this area and suggested that a combination of HLA testing and COVID-19 tests could identify high-risk individuals susceptible to COVID-19 infection and thus protect them against such severe complications by changing the vaccination strategy in high-risk populations [24] According to our knowledge, in this study the first cases of septic arthritis in the field of COVID-19 infection and its treatment are reported and given the current pandemic we expect more in the future.

In any patient with the history of COVID-19 infection specially those who have been treated with corticosteroid as one of the medications prescribed during the disease, any joint symptom specially in the hips should draw our attention to the joint infection and take the necessary measures in this regard.
